# Efficacy of the Sausage Technique in Rebuilding the Crestal Buccal Bone Thickness: A Retrospective Analysis

**DOI:** 10.3390/dj12060180

**Published:** 2024-06-12

**Authors:** Stefano Pieroni, Benedetta Miceli, Luca Giboli, Leonardo Romano, Lorenzo Azzi, Davide Farronato

**Affiliations:** 1 Private Practice, 20129 Milan, Italy; info@pieronismile.it; 2 Implant Center for Edentulism and Jawbone Atrophies, Maxillofacial Surgery and Odontostomatology Unit, Fondazione IRCCS Cà Granda Ospedale Maggiore Policlinico, University of Milan, 20122 Milan, Italy; luca.giboli@unimi.it; 3 School of Dentistry, Department of Medicine and Surgery, University of Insubria, 21100 Varese, Italy; l.romano2@studenti.uninsubria.it; 4 Department of Medicine and Technological Innovation, Unit of Oral Medicine and Pathology, ASST dei Sette Laghi, 21100 Varese, Italy; l.azzi@uninsubria.it; 5 Department of Medicine and Technological Innovation, Research Center of Innovative Technology and Engineered Biomaterial, University of Insubria, 21100 Varese, Italy; davide@farronato.it

**Keywords:** guided bone regeneration (GBR), sausage technique, bone volume, regeneration shape

## Abstract

The goal was to evaluate the efficacy of the sausage technique in reconstructing the crestal buccal bone thickness, focusing on the distribution shape of the regenerated volume. Ten implants were placed in five patients with Cawood–Howell class IV defects. A cone beam computed tomography (CBCT) was executed at T0 (before surgery). Guided bone regeneration (GBR) with the sausage technique utilized a resorbable collagen membrane, made of a 50% autologous bone and a 50% anorganic bovine bone matrix (ABBM) mixture. After 6 months, a CBCT (T1) was performed before implant placement. Using CBCT software, a plane parallel to the implant axis intersected perpendicular planes every 1.5 mm from the crest level. T0 and T1 CBCT sections were analyzed, yielding 140 measurements. Statistical analysis via SPSS revealed a significant increase in thickness (average 2.82 ± 1.79 mm). Maximum gains occurred at 4.5 mm from the coronal crest line (3.8 ± 1.51 mm). The GBR sausage technique was effective with minimal post-operative complications, yielding the biggest gain at the mid-ridge sagittal area. Within the analysis limitations, it can be assumed that the sausage technique is effective for horizontal GBR in the maxilla, but a lesser volume might be achieved at the crestal level because it seems to follow a bowed regeneration shape.

## 1. Introduction

Dental implants are widely used and represent a safe and predictable way to replace missing teeth [1,2]. For successful implantation, dental implants need to be placed inside the bone in a prosthetically driven position [3,4,5,6,7,8]. Various studies suggest that the amount of vestibular bone after implant placement plays an important role in maintaining soft tissue and preventing complications [9,10,11,12]. Moreover, the amount of crestal buccal bone seems to play a cutting-edge role in the risk of recession over the maturation period [12]. In patients with Cawood and Howell class IV defects, also known as “knife-edge” ridge, this condition is rarely satisfied and bone augmentation is required [12,13]. 

In the current literature, several methods have been suggested for the reconstruction of atrophic alveolar jaws for implant purposes, employing either one- or two-stage approaches [14,15,16,17,18,19,20,21]. These techniques include bone blocks and GBR with resorbable membranes, titanium-reinforced non-resorbable membranes, titanium meshes, or other techniques [22,23,24,25,26]. The alveolar ridge-splitting/expansion technique can be used in similar indications, allowing for a reduction in the duration of the treatment in a single surgical phase, with an increased risk of fracture and unpredictable resorption of the cortical bone component [27,28,29,30]. Historically, bone blocks were the primary option for regenerating bone defects in the alveolar ridge. Although onlay bone grafting has been associated with favorable implant survival rates, this technique involves harvesting a substantial amount of bone, often sourced from extra-oral sites, leading to increased patient morbidity [31,32,33,34,35,36,37,38]. 

In response to the limitations associated with bone block techniques, GBR emerged as an alternative for Cawood–Howell class IV atrophies [39,40]. This technique has been extensively studied and is now regarded as safe, with predictable long-term results. Research findings indicate that implants inserted concurrently with GBR procedures, employing either resorbable or non-resorbable membranes, exhibited a robust survival rate ranging from 91.9% to 92.6% observed at 12–14 years [41].

One essential principle of GBR is the use of a membrane to create a barrier, preventing the colonization from non-osteogenic tissues and maintaining space to facilitate optimal conditions for bone regeneration [42,43,44]. Clinical studies have used both non-resorbable and resorbable membranes in knife-edge ridge treatments successfully [45,46]. Non-resorbable membranes are the gold standard for the regeneration of significant defects, as they can rigidly maintain space and exclude cell populations until they are surgically removed; conversely, using resorbable membranes maintains their function within a limited period [47,48]. Moreover, the associated technique provides for the regeneration of complex shapes, such as the crestal buccal bone, as made possible by the metal reinforcement shaping. Complications from early exposure and the need for an extended second surgery are the main disadvantages when using non-resorbable membranes [47]. 

Istvan Urban proposed to fix the resorbable membranes with pins to achieve higher stability of the grafted material and to increase the volume stability [49,50]. This technique does not require a second surgical stage to remove the membrane nor, generally, the pins, and is associated with a lower rate of complications. Urban’s protocol involves grafting with a combination of autologous bone particles and deproteinized bovine bone in a 1:1 ratio. A resorbable native collagen membrane is fixed in tension through bone pins to increase the stability of the bone particles. The proposed protocol allowed for the obtainment of a higher quantity of regenerated bone with a lower rate of graft remodeling, compared to conventional GBR [51]. Nevertheless, the sausage technique shows positive outcomes in the literature, yet the optimal indications to support its choice seem unclear [52,53,54,55]. The shape of the graft appears to depend on the tension of the membrane, and this may represent a partial limitation when the goal is to rebuild the buccal bone at the crestal level. 

The primary aim of this analysis is to assess the efficacy of the GBR ‘sausage technique’ in reconstructing the crestal buccal bone thickness in patients requiring horizontal bone augmentation before implant placement. The research focuses on analyzing the shape and extent of the regenerated bone volume, particularly in the buccal crest, to assess the technique’s efficacy. This includes examining the augmentation patterns at various distances from the crestal edge. 

As a secondary objective, the study investigates the incidence of both early and late complications associated with the procedure.

## 2. Materials and Methods

In this analysis, five patients (two women and three men with an average age of 54.4 years, ranging from 38 to 68) were enrolled from March 2017 to the end of July 2018 in a private dental practice in Milan. All patients required horizontal augmentation using guided bone regeneration (GBR) according to the sausage technique for subsequent implant placement. They presented with knife-edged ridges (Cawood–Howell class IV), including both maxillary edentulous ridges and a mandibular ridge. Six areas of regeneration were identified (5 in the maxilla and 1 in the mandible), and a total of ten measurement sites were established across the six surgical sites for the inserted dental implants, as shown in Table 1.

Implants were placed in the first sextant (two implants), in the second sextant (six implants), and in the fourth sextant (two implants), as shown in Table 2.

### 2.1. Inclusion Criteria 

The criteria for inclusion of participants in the analysis were age over 18, good oral hygiene (Plaque Index < 10%, Bleeding Score < 25%), edentulous site healed from any extractions for at least three months, and good general health status. 

### 2.2. Exclusion Criteria 

The criteria for exclusion of participants from the analysis were patients requiring vertical bone augmentation; uncompensated periodontal pathology; special or uncompensated pathologies; intake of anticoagulant drugs, glucocorticoids or bisphosphonates; history of irradiation in the head and neck area; smokers of >five cigarettes per day; excessive alcohol consumptions; and pregnant or lactating patients.

### 2.3. Clinical Protocol 

The preparation protocol for surgery included the following steps for all patients: This regimen commenced with an intensive oral hygiene session conducted by a dental professional one week prior to the surgical intervention to minimize any potential sources of infection. Patients were then instructed to commence a rigorous oral rinsing routine, employing a 0.12% chlorhexidine mouthwash. This regimen was to be followed twice daily for one week leading up to the operation and continued post-operatively until the removal of sutures. Furthermore, an immediate pre-surgical rinse was performed to reduce oral microbial load. The antibiotic prophylaxis was carefully prescribed, with patients commencing a course of amoxicillin and clavulanic acid (Augmentin, GlaxoSmithKline, Brentfor, UK) at a dosage of 1 g every 12 h. This course was initiated one day before the operation and extended for a total of seven days. Additionally, an anxiolytic premedication consisting of delorazepam (En, Mylan, Italy) was administered 30 min prior to the surgical procedure to ease patient anxiety. This was complemented by a preoperative anti-inflammatory regimen and thorough perioral skin disinfection with iodopovidone solution (Betadine, Viatris, Milan, Italy). Anesthesia was induced using a plexic local anesthesia with a composition of 4% articaine and epinephrine at a concentration of 1:100,000 (Citocartin 100, Molteni Dental, Milan, Italy) to ensure patient comfort and vasoconstriction to limit bleeding during the procedure.

The enrolled patients were treated with bone regeneration using the GBR technique according to the “sausage technique”. The procedure commenced with the administration of local anesthesia to the affected area to ensure a pain-free experience. A crestal incision was then performed using a cold blade scalpel, from which two flaps were raised, delineating the area of the bone defect, which was subsequently confirmed.

A double-layer resorbable membrane derived from natural collagen (Bio-Gide Resorbable Bilayer Membrane, Geistlich Pharma, Wolhusen, Switzerland) was chosen. It was positioned over the crest to cover the entire grafting area and securely fixed to the apical region of the residual alveolar crest with pins. Autologous bone particulate was harvested. The grafting compound was prepared, consisting of a 50% autologous bone and 50% Anorganic Bovine Bone Mineral (ABBM) mixture (Bio-Oss, Geistlich Pharma).

To create the “sausage”, the grafting material was inserted into the pocket formed between the bone crest and the resorbable membrane, secured with the first set of pins. Additional pins were subsequently applied. The advancement of flaps through periosteal incisions and their subsequent suturing allowed for primary intention closure.

Post-operative instructions were carefully detailed to all patients, emphasizing the application of cold compresses in 15 min intervals to mitigate swelling, adherence to a diet of soft and cold foods to minimize mechanical stress on the surgical site, avoidance of vigorous physical activities that could disrupt the healing process, and refraining from wearing mobile prostheses in the operated area to prevent undue pressure. Sutures were meticulously removed at the two-week mark, following surgery.

After a recovery period of six months, the patients underwent a second surgery for implant placement. During this period, the degree of bone neoformation was assessed. The implants were inserted using the two-stage technique, whereby at the time of insertion, the implants were submerged without being subjected to any contact with the oral environment or any prosthetic load.

After 4 months of osteointegration, a third surgery was performed: the mucosa was reopened to connect the implant to the prosthetic components. In cases where it was deemed necessary, during the reopening phase, a restoration of keratinized mucosa was performed using epithelial connective tissue grafts or apically repositioned flaps.

Complications in bone graft healing, such as membrane exposure, infection, and morbidity associated with the harvest site were recorded. 

### 2.4. Data Collection

To study the difference in bone volume detectable after the regenerative surgery, a CBCT (cone beam computed tomography) was performed for all patients before the first surgery and before implant placement (at 6 months after the first surgery). 

This allowed for the study of the difference in bone volume detectable following the regeneration intervention. All radiographic examinations were performed in the same center and with the same machine. The settings of the radiographic machine were not necessarily the same for each patient and each CBCT scan: the kilovoltage was kept fixed and stable for each image, while the exposure time and optical density (mAs) varied from time to time. Radiographic images were therefore considered comparable following calibration of the measurement tool used.

**Figure 1:**
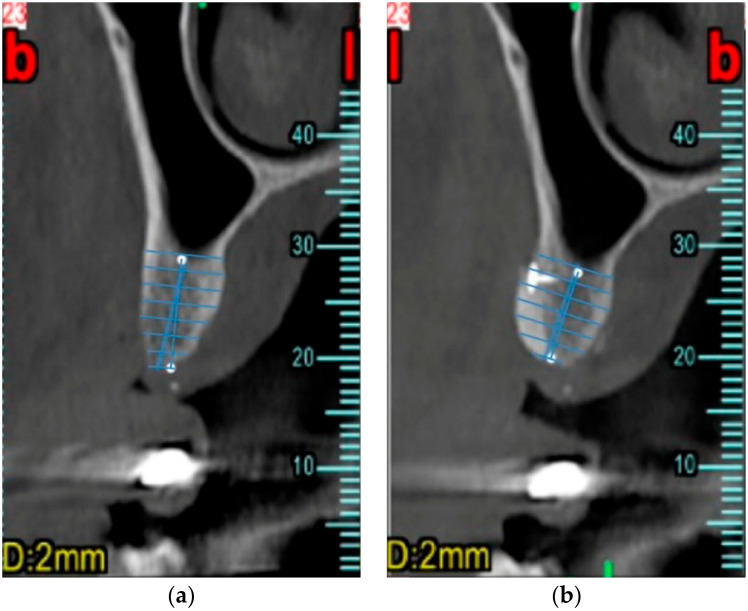
Example of bone thickness measurement. A CBCT section of the crest was selected for each implant site. A line 13 mm in length was drawn along the direction of the ideal implant axis. Perpendicular lines to the implant axis were then identified, marking the variation in bone crest thickness every 1.5 mm. The length of each of these lines was measured. (**a**) Measurement at baseline (T0); (**b**) Measurement after 6 months (T1).

CBCT sections were created and processed using the Soda PDF software (Soda PDF 11, LULU Software, Montreal, QC, Canada) and measurements were made using ImageJ software (ImageJ 1.52, National Institutes of Health (NIH), Bethesda, MD, USA), to compare corresponding sections of the ridges before and after surgery. Once the outlines of the cortical bone profile were defined, a section was selected for each implant site. CBCT at T0 and T1 were superimposed manually using anatomically stable landmarks; then, measurements at various heights were performed using the most crestal point as 0. Lines perpendicular to the ideal implant axis were then identified, identifying changes in bone ridge thickness every 1.5 mm, as shown in Figure 1. The length of each of these lines was measured and recorded in a table. By comparing the corresponding thickness lines between the pre-operative and post-operative sections, it was possible to evaluate the bone augmentation achieved. 

### 2.5. Data Analysis

Throughout the study, a total of 205 data points were collected, and out of these, only 140 were suitable for statistical analysis. The exclusion of data was due to different crest heights or to the impossibility of obtaining 10 measurements of the thicknesses. The SPSS Statistics program was used for data processing. Median values, mean values before and after the interventions, median ∆, mean ∆, interquartile ranges (IQRs), and standard deviations (SDs), with a 95% confidence interval (CI) were defined. Comparisons between the measurements at time zero (T0) and those after the operations were made with the non-parametric Wilcoxon test for paired data to detect changes in the volumetric dimensions of the bone ridges. Values with a *p* ≤ 0.05 were considered significant.

## 3. Results

At six months after surgery, there were no graft failures, resulting in a 100% success rate, as shown in Table S1 (Supplementary Materials).

Furthermore, no cases of membrane exposure or other biological or surgical complications were reported. Some patients experienced minimal post-operative distress, including swelling, pain, and bruising, with no discomfort persisting beyond two weeks.

Descriptive statistics, including the mean, standard deviations (SD), median, and interquartile ranges (IQRs), for the measurements before and after the surgery are presented in Table 3 and Table 4, respectively.

Table 3 outlines the baseline descriptive statistics, encompassing mean, median, standard deviations (SDs), and interquartile ranges (IQRs), along with the standard deviations of the pre-surgical measurements, all expressed in millimeters.

Table 4 provides similar descriptive statistics for the 6-month post-surgical measurements, detailing the changes observed in the bone ridges’ volumetric dimensions over the study period.

The changes in bone thickness (Δ values) are comprehensively tabulated in Table 5. The Δ values were found to be statistically significant according to the Wilcoxon test. In fact, the Wilcoxon signed-rank test conducted on the Δ values demonstrates statistical significance, with a *p*-value < 0.001, which is indeed less than 0.001. This confirms the substantial change in bone thickness from baseline to the 6-month measurement. 

The average increase in bone thickness is 2.82 ± 1.79 mm. The average thickness gains obtained are 1.19 mm at 0 mm from the crest, 2.70 mm at 1.5 mm, 3.48 mm at 3 mm, 3.80 mm at 4.5 mm, 3.49 mm at 6 mm, 2.76 mm at 7.5 mm, and 2.34 mm at 9 mm, as shown in Table 4.

**Figure 2:**
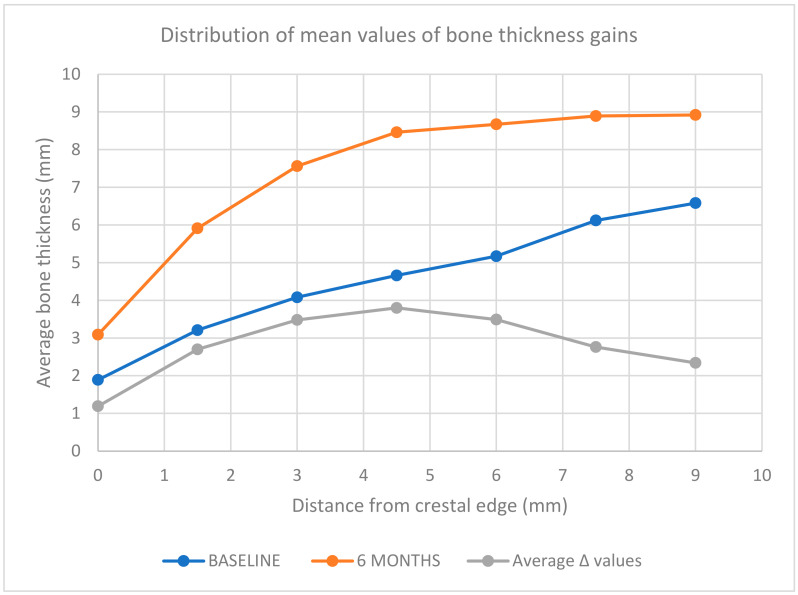
Distribution of mean values of bone thickness gains. The average bone thickness gain is represented at different distances (mm) from the crestal edge: T0 (blue line), T1 (orange line), and the average regenerated thickness (grey line) are compared.

The greatest increase in bone thickness occurs at 4.5 mm from the most coronal point of the crest, reaching a peak difference of 3.80 ± 1.51 mm in average thickness. These findings are graphically depicted in Figure 2.

**Figure 3:**
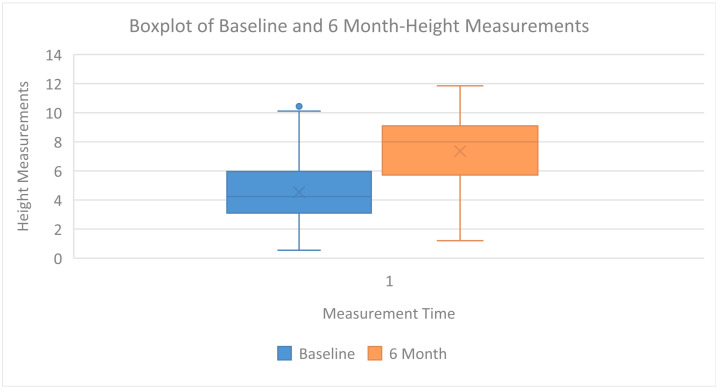
The boxplot displays height measurements at baseline and 6 months, with medians of 4.24 and 7.99, respectively. The interquartile range (IQR) at baseline is 2.70, expanding to 3.28 at 6 months, indicating increased variability. Points outside the whiskers represent outliers. This boxplot compares central tendency and variability across two time points.

In the boxplot below (Figure 3), the distribution of height measurements collected at baseline and after 6 months are visually represented. For baseline measurements, the median is depicted at 4.24, with an interquartile range (IQR) of 2.70. At the 6-month mark, the median increases to 7.99, with an IQR of 3.28, reflecting a general increase in the measurements over time. 

## 4. Discussion

This analysis assesses the effectiveness of the guided bone regeneration (GBR) ‘sausage technique’ across six surgical sites involving five patients, for horizontal bone augmentation prior to dental implant placement. It scrutinizes the outcomes at ten distinct measurement sites, with the objective of reconstructing the ridge morphology for implant support. The data obtained reveal that the increase in bone thickness at six months after the procedure occurred in all the edentulous ridges analyzed. In the present analysis, the mean horizontal bone gain was 2.82 mm (±1.79 mm). 

In this study, the data reveal a noteworthy increase in the median height from the baseline value of 4.24 to 7.99 at the 6-month mark. This substantial increase of 3.75 units in the median height suggests a significant growth trend over the measured time frame.

The statistical analysis, as per the Wilcoxon signed-rank test, yields a *p*-value of less than 0.001. This low *p*-value strongly suggests that the observed changes in bone thickness are not due to random variation, but rather are attributable to the intervention effect. The substantial nature of the changes is thus statistically affirmed, indicating that the bone regeneration treatment has had a significant impact on bone thickness.

Even considering the limitations of this analysis, the present numbers are comparable with the current literature [49,50,51,52,53,54]. A study by Meloni obtained similar results after GBR surgery with resorbable membranes and the same type of graft material at one year after implant loading [53,54]. The results of the present analysis are slightly inferior to those obtained by Urban [49,50], Meloni [53,54], and Kim K.M. [52], as in this case, an average of bone thickness measurements taken every 1.5 mm along the entire alveolar ridge was performed. 

These measurements allowed for the evaluation of interesting data. An aspect that recurs in all the samples analyzed is that the largest bone regeneration is located at the mid-ridge area, considering a sagittal view of the GBR procedure. The greater increase in volume is noted between 4.5 and 6 mm from the most coronal part of the ridge. Conversely, in the crestal zone, the bone increase is modest.

The explanation for this observation could be a greater compression at the crestal levels to which the graft is subjected once the resorbable collagen membrane is placed, compared to the central area, where this compression is lower. Therefore, the graft will tend to be placed in this central area. 

This could be a limited indication of this technique. Buser et al. investigated the ideal amount of hard tissue required around dental implants and found that the thickness of the buccal bone at the implant site played a fundamental role in the esthetic predictability of the rehabilitation [7]. A recent study revealed the importance of the buccal bone thickness at the implant placement as a key influence on the stability of the facial gingival margin over a maturation time of three years [12]. In this study, a buccal bone thickness to the implant of at least 2 mm is necessary to obtain stable long-term results and a good soft-tissue esthetic.

In the considered sample, the mean thickness at 1.5 mm from the most coronal point of the crest at T0 was 3.21 mm; thus, the mean thickness at 1.5 mm at T1 was 5.91 mm. Given an average implant diameter of 4 mm and assuming an implant position leaving 1 mm palatally to the implant, the residual buccal bone thickness was 0.91 mm and was thus insufficient. 

If GBR protocols have to achieve not only implant stability but also pursue the recommended bone thickness, the proposed technique might not be recommended for the regeneration of class IV defects with a crestal thickness at 1.5 mm smaller than 4.3 mm. 

Even if a recent study has reported positive outcomes, the application of the technique for slight vertical increments necessitates evaluation in longer follow-ups to assess true effectiveness [52]. Therefore, because one of the major limitations of the sausage technique is the crestal height augmentation, non-resorbable membranes are still the most predictable choice as confirmed by Nikolaos K Soldatos et al. in a systematic review of the literature [47]. For these reasons, vertical bone regeneration was excluded from this evaluation.

The analysis revealed consistent favorable bone density at the six-month mark post-healing, underscoring the effectiveness of the ‘sausage technique’ in fostering bone regeneration conducive to implant stability. A clear subperiosteal cleavage plane was identified, with no clinical observation of evident connective infiltrations. Consistency between the radiographic view of the graft and the clinical perception was found in all sites, indicating an acceptable bone regeneration.

The newly formed bone demonstrated clinically dense integration with the surrounding osseous tissue, which facilitated the surgical maneuvers during implant placement and ensured optimal stability of the implants.

The regenerated bone was found to be of satisfactory quality and volume for the successful support of the implants.

Concerning soft tissue management, in cases where it was deemed necessary, efforts were made to optimize soft tissue to ensure a minimum of 2 mm of keratinized mucosa in the buccal area [56]. This was achieved through the use of epithelial connective tissue grafts or apically repositioned flaps, enhancing the soft tissue profile for the long-term success of the implants.

In the present analysis, any complications related to the procedure were investigated. Only minimal post-operative discomfort was reported. The swelling was significant in the majority of cases but subsided in the initial days and disappeared after 7 days. Pain was primarily associated with tension due to swelling. However, in this analysis, no major complications occurred. Complications such as membrane exposures can be treated with simple pharmacological solutions, such as rinses with chlorhexidine 2% mouthwash twice a day, as indicated in the post-surgical prescriptions of this analysis and as suggested by Meloni’s studies [53,54]. If other techniques using non-resorbable membranes were used, the only solution would have been the early removal of the membrane itself, compromising graft healing and often preventing adequate bone volume from being obtained. 

Furthermore, the composition of the graft with 50% ABBM reduces the amount of autologous bone taken and, consequently, the incidence of complications at the bone-harvesting site is reduced. The present technique shows a low risk of morbidity. Therefore, despite minimal post-operative discomfort, the absence of major complications supports the benefit of this type of procedure.

This analysis presents several limitations. The sample size is limited, and the follow-up period of only six months could be considered short. Additionally, the outcomes of a single operator were examined, which could introduce a confounding factor as the examination did not exclude operator-dependent variables. Moreover, considering the differences in bone quality and vascularity between the maxilla and mandible, the limited number of regenerated sites—predominantly in the maxilla—may not provide a comprehensive assessment of the anatomical impact on bone regeneration. This factor is particularly relevant given the mandible’s lower vascularity, which could potentially affect the success of regeneration and implant integration. Therefore, future studies may require not only a larger number of surgeries but also a balanced examination of both anatomical sites, monitored over a more extended follow-up period and involving multiple surgeons to minimize individual variability and confirm the present findings. This extended period would allow researchers to assess not only the immediate post-operative success but also the stability and longevity of the bone regeneration results. Such data are crucial for confirming the efficacy of the procedure over time and for evaluating any late-onset complications or failures.

## 5. Conclusions

The analysis revealed a successful increase in bone thickness in all the patients treated with the“sausage technique“ and the absence of major complications. According to the literature, the sausage technique is an effective and reliable approach for horizontal bone augmentation. 

Within the limits of the current analysis, the results highlight that the most substantial bone regeneration occurred at the mid-ridge sagittal area, indicating a potential limitation in the application of this technique. 

A long-term assessment of implant survival and bone remodeling is still necessary to gauge the predictability of this technique. Additionally, the distribution of bone augmentation in relation to the crestal edge may be investigated in multicentric prospective studies to eliminate operator-dependent variables.

## Figures and Tables

**Table 1:** Summary of patient demographics and surgical details.

Category	Total	Details
Patients treated	5	Two women and three men, average age 54.4 years
Regeneration areas	6	5 in the maxilla, 1 in the mandible
Measurement sites	10	Distributed across the 6 surgical sites
Dental implants inserted	10	Distributed across the measurement sites

**Table 2:** Number of implants per sextant.

Sextant	Number of Implants
I	2
II	6
III	0
IV	2
V	0
VI	0

**Table 3:** BASELINE—Mean, standard deviations, median, and interquartile range of the pre-surgical section (expressed in mm).

Height Measurement	Average Value	DS	Median	Interquartile Range
0	1.89	1.17	1.52	1.09
1.5	3.21	1.25	3.38	1.54
3	4.08	1.46	4.22	1.53
4.5	4.66	1.54	4.78	2.16
6	5.17	1.64	5.23	2.86
7.5	6.12	2.38	5.94	3.27
9	6.58	2.64	6.11	3.95

**Table 4:** 6 MONTHS—Mean, standard deviations, median, and interquartile range of the post-surgical section (expressed in mm).

Height Measurement	Average Value	DS	Median	Interquartile Range
0	3.09	1.48	2.98	1.60
1.5	5.91	1.56	6.05	1.86
3	7.56	1.76	7.57	1.78
4.5	8.46	1.64	8.43	2.35
6	8.67	1.38	9.03	1.48
7.5	8.89	1.17	9.00	1.17
9	8.92	1.54	8.89	1.85

**Table 5:** Mean, standard deviations, median, and interquartile range of ∆ values (expressed in mm).

Height Measurement	Average Value	DS	Median	Interquartile Range
0	1.19	1.66	0.77	1.12
1.5	2.70	1.59	2.43	0.96
3	3.48	1.74	2.63	2.31
4.5	3.80	1.51	3.18	2.01
6	3.49	1.06	3.41	1.60
7.5	2.76	2.30	1.96	3.69
9	2.34	2.69	1.42	4.36

## Data Availability

The data presented in this study are available on request from the corresponding author.

## Supplementary Materials

The following supporting information can be downloaded at https://www.mdpi.com/article/10.3390/dj12060180/s1, Table S1: Crestal thickness measurements for each section for each patient (expressed in mm).
