# Supplementary material for: Efficacy of the Sausage Technique in Rebuilding the Crestal Buccal Bone Thickness: A Retrospective Analysis
Source: Dent J (Basel). 2024 Jun 12;12(6):180. doi: 10.3390/dj12060180 (PMC11202426; doi:10.3390/dj12060180)
Supplement: Supplementary file 1 [file dentistry-12-00180-s001.zip › dentistry-2944924-supplementary.pdf]

Supplementary materials

Table S1 - Crestal thickness measurements for each section for each patient (expressed in mm)

	Patient A				Patient B		Patient C				Patient D				Patient E					
	Pre		Post		Pre	Post	Pre		Post		Pre		Post		Pre			Post		
	Site 1	Site 2	Site 1	Site 2	Site 1	Site 1	Site 1	Site 2	Site 1	Site 2	Site 1	Site 2	Site 1	Site 2	Site 1	Site 2	Site 3	Site 1	Site 2	Site 3
Sextant	II	II	II	II	II	II	II	II	II	II	IV	IV	IV	IV	II	I	I	II	I	I
0.0	2.56	4.46	3.06	2.99	0.55	1.39	1.5	3.16	2.66	3.86	1.55	1.54	5.62	5.24	1.21	1.24	1.15	1.86	1.2	2.97
1.5	3.78	4.27	6.63	5.75	0.94	3.51	4.38	4.49	6.41	6.35	2.97	2.72	8.06	8.33	4.27	1.56	2.74	4.61	4.42	5.04
3.0	5.27	5.31	7.87	7.59	1.5	4.15	5.87	5.04	8.72	7.54	4.22	3.55	9.57	10.21	4.22	1.79	4.06	5.98	7.56	6.45
4.5	6.35	5.13	8.43	8.41	2.3	5.39	7.04	5.81	10.03	8.42	5.08	4.45	10.09	10.76	3.15	2.84	4.48	7.00	8.99	7.09
6.0	6.69	4.22	8.96	9.13	3.49	5.61	7.35	6.24	10.31	9.1	6.43	6.45	9.64	10.05	3.23	3.5	3.86	7.98	8.2	7.69
7.5	7.32	4.26	8.35	9.09	5.36	6.28	8.72	6.53	10.2	8.97	7.53	10.44	8.86	9.83	3.56	4.19	3.32	9.04	10.22	8.01
9.0	7.63	4.22	7.61	8.71	5.43	6.37	10.11	6.79	9.86	8.69	8.64	10.75	9.06	10.13	3.84	5.1	3.26	9.34	11.85	7.59
10.5	7.25	4.74		7.72	5.34	6.1	11.69	7.61	11.21	9.02	8.78	10.6	8.95	10.11	4.08	6.38	4.17	10.06	12.87	6.95
12.0		5.47		6.03	5.48	5.6	13.73	8.13	12.54	10.22	8.93	10.59	9.16	10.16	4.98	8.25	5.3	7.75	13.15	7.88
13.5		7.03		5.81	5.69	5.55		9.4	13.6	11.8	9.1	10.59	9.25	10.18	6.69	10.81	6.64	10.64	13.09	9.65
